# Cultural Adaptation and Validation of the Pelvic Floor Distress Inventory Short Form (PFDI-20) and Pelvic Floor Impact Questionnaire Short Form (PFIQ-7) Portuguese Versions

**DOI:** 10.3390/healthcare13233136

**Published:** 2025-12-02

**Authors:** Inês Branco, Mariana Ferreira, Ana Pacheco, Clara Ferreira, Vera Baldaia Dias, Anabela Correia Martins

**Affiliations:** 1 Coimbra Health School, Polytechnic University of Coimbra, Rua 5 de Outubro, 3045-043 Coimbra, Portugal; a2019139123@estescoimbra.pt (I.B.); marianadferreira@estesc.ipc.pt (M.F.); 73312@ulsts.min-saude.pt (A.P.); claraferreira@ulsts.min-saude.pt (C.F.); 72051@ulsts.min-saude.pt (V.B.D.); 2 Tâmega e Sousa Local Health Unit, Avenida do Hospital Padre Américo, Nº 210, 4560-136 Penafiel, Portugal; 3 H&TR-Health & Technology Research Center, Coimbra Health School, Polytechnic University of Coimbra, Rua 5 de Outubro, 3045-043 Coimbra, Portugal; 4 Center for Rehabilitation Research, Health School, Polytechnic of Porto, Rua Dr. António Bernardino de Almeida, 4200-072 Porto, Portugal

**Keywords:** fecal incontinence, pelvic floor dysfunctions, pelvic organ prolapse, PFDI-20, PFIQ-7, psychometric validation, quality of life, urinary incontinence

## Abstract

**Introduction:** Throughout life, the characteristics of a woman’s pelvic floor change due to physiological changes, including pregnancy, childbirth and menopause. These changes can predispose them to pelvic floor dysfunction. **Objectives:** To develop a linguistically and psychometrically adapted Portuguese (European) version of the Pelvic Floor Distress Inventory (PFDI-20) and Pelvic Floor Impact Questionnaire (PFIQ-7), for assessing symptoms and quality of life in women with pelvic floor dysfunction. **Methods:** This cross-cultural study used a translation method, followed by an assessment of the validity and reliability of the instruments. The Portuguese versions of the PFDI-20 and PFIQ-7 were completed by 287 women (33.47 ± 8.2 years). To assess reliability, internal consistency was evaluated using Cronbach’s alpha (CA). Descriptive statistical analysis was applied for sociodemographic and clinical characterization, as well as questionnaire scoring. Spearman’s correlation (r) and Student’s *t*-test were used to analyze criterion and construct validity. **Results:** The Portuguese versions of PFDI-20 and PFIQ-7 were effectively translated and adjusted, revealing excellent internal consistency, as reflected in Cronbach’s alpha values of 0.853 for PFDI-20 and 0.937 for PFIQ-7. No Ceiling Effect was observed, while a Floor Effect was identified in both Portuguese versions of the PFDI-20 (5.2%) and PFIQ-7 (41.5%). Significant correlations were established between the instruments and five questions. **Conclusions:** The Portuguese versions of the PFDI-20 and PFIQ-7 showed adequate psychometric characteristics and are valid for use in the Portuguese population.

## 1. Introduction

The pelvic floor is composed of bones, ligaments, and muscle structures that encompass the lower region of the abdominopelvic cavity. Supporting pelvic organs, controlling sphincters, facilitating bladder and bowel activities, and ensuring sexual and reproductive functions are among their primary roles. Pelvic floor muscles are responsible for maintaining continence, allowing visceral urination, ensuring optimal sexual and reproductive function, and providing lumbopelvic stability [1,2]. Over the years, physiological changes related to pregnancy, childbirth, and menopause, as well as aging and lifestyle factors, contribute to the development of pelvic floor dysfunctions in women. These conditions, resulting from hormonal, anatomical, and biomechanical alterations, are highly prevalent and represent a significant public health concern due to their substantial impact on the quality of life of millions of women worldwide [2,3,4,5]. The International Continence Society categorizes PFD into five domains related to the lower urinary tract (urinary incontinence, frequency, urgency, flow or difficulty in urination), bowel function (fecal incontinence, urgent rectal prolapse, obstipation), vaginal symptoms (pelvic organ prolapse), sexual function (dyspareunia and vulvodynia), and chronic pelvic pain [5].

PFD emerges from weakness in the supportive tissues of the pelvic floor, encompassing conditions such as urinary incontinence (UI), pelvic organ prolapse (POP), fecal incontinence (FI), sexual dysfunction, and other urogenital symptoms, along with lower urinary and gastrointestinal tract emptying issues. While these symptoms can occur independently, they often coexist. The severity of these symptoms can be exacerbated by factors such as childbirth, pregnancy, age, obesity, constipation, chronic cough, pelvic surgery, physically demanding activities stressing the pelvic floor, and even genetic predisposition [3,5,6,7,8]. Although more common in women, pelvic floor dysfunctions are also prevalent in both sexes [8,9].

While not fatal, PFD has biopsychosocial consequences, affecting mood, mentality, self-esteem, and significantly reducing the quality of life, with implications for social, domestic, and sexual aspects [10]. This chronic condition is associated with diminished quality of life and physical, social, and psychological well-being [6,7,11,12]. Consequently, these factors can lead to increased likelihood of symptoms like depression, anxiety, frustration, and nervousness [11]. In terms of physical health, these symptoms lead to evident impacts, including perineal skin rashes, pressure ulcers, and urinary and uterine infections [13].

Due to a lack of awareness about the topic, pelvic floor dysfunctions tend to be undervalued. People often hesitate to seek healthcare professionals as they consider these issues “normal.” Since such matters are not openly discussed within communities, even highly educated individuals may remain uninformed about these health conditions [2,5,13].

To comprehensively assess this condition, it is important to evaluate movement patterns, physical and psychological states, beliefs about the condition, consequences for daily life aspects such as work, physical activity, household tasks, sexuality, social and family life, and contextual factors (external and personal) like motivation and adherence [14].

The prevalence of this health condition varies from 5% to 69% depending on the studied population, with most studies indicating an average range of 25% to 45% [11,15]. The Portuguese Society of Gynecology estimates that around 50% of adult females suffer from UI, but only 25% to 61% seek treatment. The overall prevalence of POP, based on clinical evaluation in menopausal women above 50 years, is approximately 40%. It is also mentioned that between 2000 and 2012, around 46,819 hospital discharges in public hospitals were registered with a primary or secondary diagnosis of POP in Portugal [16]. Regarding FI, a prevalence of up to 18% is estimated for the general population, which can reach 50% in institutionalized patients. Its prevalence increases with age and is distributed scalarly in both sexes [17]. Regarding sexual disorders, it is estimated that 40% of women experience this condition during their early reproductive years [5]. Despite the high prevalence of PFD, scientific evidence and research on managing these conditions are extremely limited [8].

Assessing the quality of life in individuals with pelvic floor disorders (PFD) is essential for developing effective treatment plans, and evaluating subjective perception is therefore crucial. In this context, the scientific and professional community in Portugal has expressed interest in the availability of a Portuguese version of these questionnaires for use in the field of pelvic health.

In 2005, Barber, in the USA, developed two short versions of previously designed specific questionnaires. These psychometrically valid and reliable instruments assess how PFD affects women’s health-related quality of life. The first, Pelvic Floor Distress Inventory (PFDI-20), evaluates symptoms caused by pelvic floor dysfunctions and their impact, while the second, Pelvic Floor Impact Questionnaire (PFIQ-7), measures the impact of PFD symptoms on quality of life. These questionnaires have been translated into multiple languages, including French, Spanish, Turkish, Japanese, Danish, Dutch, Brazilian Portuguese among others, facilitating cross-population comparisons and multinational studies [18]. Although Brazilian and European Portuguese share a linguistic basis, cultural and regional differences can affect symptom interpretation and health terminology. Therefore, the European Portuguese version underwent rigorous cultural validation to ensure conceptual equivalence and clarity for the target population [19].

The primary objective of this study was to perform the cultural adaptation and psychometric validation of the PFDI-20 and PFIQ-7 questionnaires for use in the European Portuguese population. Specifically, the study aimed to translate and adapt these instruments and to evaluate their validity and reliability in a sample of Portuguese women. This process was necessary, as no validated versions of these instruments for European Portuguese were available prior to this research. The European Portuguese validation of the PFDI-20 and PFIQ-7 enables accurate screening, diagnosis, and monitoring of pelvic floor disorders in Portuguese-speaking women. These culturally adapted tools facilitate patient-reported outcome tracking in both clinical practice and research, supporting individualized care and inclusion in international studies [20].

## 2. Materials and Methods

This study was conducted in two phases: the first phase involved the translation of the instruments into European Portuguese, and the second phase comprised the evaluation of their psychometric properties.

### 2.1. PHASE 1

The translation and cross-cultural adaptation of the PFDI-20 and PFIQ-7 questionnaires was carried out following the recommendations of the Scientific Advisory Committee of the Medical Outcomes Trust [21]. Initially, both instruments were independently translated into European Portuguese by two bilingual translators, whose native language was Portuguese, ensuring both lexical and cultural equivalence. Subsequently, each translation underwent a back-translation process. The translated versions were then reviewed in a preliminary academic consensus panel composed of six experts with extensive experience in instrument translation and back-translation, as well as in pelvic floor disorders. Panel members were instructed to assess semantic equivalence, and full consensus was achieved for all items. This process resulted in preliminary European Portuguese versions of the PFDI-20 and PFIQ-7. These versions were subsequently tested in a pilot group of 10 Portuguese women, native speakers, with diverse educational background. After completing the questionnaires, participants provided feedback to identify and address any potential issues. No concerns regarding clarity, comprehension, cultural relevance, or appropriateness of the wording were reported.

### 2.2. PHASE 2 Validation Study

#### 2.2.1. Study Design

Between November 2022 and May 2023, a cross-sectional study was undertaken to assess the psychometric properties of the European Portuguese adaptations of PFDI-20 and PFIQ-7.

The study was approved by the Ethics Committee of the Polytechnic Institute of Coimbra (CEIPC) in Portugal (Registration code: 170_CEIPC_2022). Permission for translation was also obtained from the original authors of the questionnaires. Participants were informed about the study’s objective and invited to participate. All participants provided written informed consent prior to enrollment, and data were handled confidentially in line with data protection regulations.

Women with Portuguese nationality, fluent in European Portuguese, and aged 18 years or older, with or without symptoms of pelvic organ prolapse (POP), urinary incontinence (UI), or fecal incontinence (FI), were included in the study. Exclusion criteria comprised women younger than 18 years, those who were cognitively unable to complete self-administered questionnaires, or those who did not provide prior informed consent.

#### 2.2.2. Participants

The sample size of the study was determined following the recommendation to have at least 10 respondents per item in the questionnaires, according to the Consensus-based Standards for the Selection of Health Measurement Instruments (COSMIN) [22]. Thus, the estimated sample size was 200. A convenience sample of 287 women were selected to the study.

#### 2.2.3. Measurement Instruments

The PFDI-20 consists of three subscales: Pelvic Organ Prolapse Distress Inventory (POPDI-6), Colorectal-Anal Distress Inventory (CRADI-8), and Urinary Distress Inventory (UDI-6). Response options range from 0 (not present) to 4 (quite a bit). The average score of all questions is calculated (between 0 and 4) by multiplying them by 25 to obtain the total score for each subscale (range 0 to 100). Missing items are handled by using the mean of answered items. The total score is obtained by summing the three subscales (range 0 to 300). Higher scores indicate more pelvic floor dysfunction symptoms [23,24].

Comprising twenty-one questions, the PFIQ-7 evaluates the quality of life experienced by women, considering how pelvic floor symptoms (related to the bladder, vagina/pelvis, and intestine) affect their physical, social, and emotional well-being [11]. It is divided into three subscales, each with seven questions: Pelvic Organ Prolapse Impact Questionnaire (POPIQ-7), Colorectal-Anal Impact Questionnaire (CRAIQ-7), and Urinary Impact Questionnaire (UIQ-7). Each question’s score ranges from 0 (not at all) to 3 (quite a bit). Within each subscale, the average score of all questions is multiplied by 33.3 to obtain the total score (range 0 to 100). The total score is obtained by summing the scores of the three subscales (range 0 to 300) [23,24].

Translating a measurement instrument to another language involves different levels of equivalence, both lexical (language) and cultural in nature. The Portuguese versions of the PFDI-20 and PFIQ-7 were developed through a translation-back-translation process, following the questionnaire translation recommendations proposed by the Scientific Advisory Committee of the Medical Outcomes Trust [20].

The Portuguese version translators, native speakers of Portuguese with high proficiency in English, independently translated the PFDI-20 and PFIQ-7. Both translations were reviewed by a consensus panel of four physiotherapists. This preliminary version was subjected to blind back-translation into English by two independent bilingual experts. All inconsistencies between the obtained English version and the original were analyzed and addressed through the consensus panel to ensure the resulting instruments were both comprehensible and conceptually consistent with the originals. While maintaining consistency with the original instrument, some expressions or phrases were slightly adjusted to ensure complete understanding by the Portuguese population.

The initial Portuguese versions of the PFDI-20 and PFIQ-7 were filled out by six native Portuguese speakers who were proficient in the language. They provided feedback to identify and correct potential difficulties in completion, comprehension, cultural adaptation, and we obtained the final Portuguese versions of the questionnaires.

Reliability was evaluated by assessing internal consistency using Cronbach’s alpha. Test–retest reliability was not performed, as the study employed a cross-sectional design without the possibility of reassessing participants at a later time.

In this study, content, criterion, and construct validities were evaluated. Content validity assesses whether the questionnaires are understood and whether all important and relevant items were included. This was achieved through expert panel analysis and clarification interviews on content and terminology used. Floor and Ceiling Effects were also calculated. We identified an issue if more than 15% of participants scored at the extreme ends, either the maximum or minimum possible values [20,24].

Criterion validity predicts the outcome in a specific situation and was assessed through the comparison of the questionnaires among themselves and with five questions (Q1–Q5) related to the perception of health, physical fitness, ability to perform specific activities, and mental/emotional well-being, namely: Q1—“In general, would you say your health is?”; Q2—“Does your health now limit you from climbing several flights of stairs? If so, how much?”; Q3—“ During the past 4 weeks, have you had any of the following problems with your work or other regular daily activities as a result of your physical health?” Accomplished less than you would like; Q4—“ During the past 4 weeks, have you had any of the following problems with your work or other regular daily activities as a result of any emotional problems (such as feeling depressed or anxious)? Accomplished less than you would like.”; Q5—“ During the past 4 weeks, how much of the time has your physical health or emotional problems interfered with your social activities (like visiting friends, relatives, etc.)?”. With the following response options: Q1—“Excellent”, “Very Good”, “Good”, “Fair”, “Poor”; Q2—“Yes, limited a lot”, “Yes, limited a little”, “No, not limited at all”; Q3–Q5: “All of the time”, “Most of the time”, “A good bit of the time”, “Some of the time”, “None of the time”. Assuming positive associations between the results of the Portuguese versions of the PFDI-20 and PFIQ-7 and Q1, Q2, Q3, Q4, and Q5.

Construct validity was evaluated through several hypotheses we established, namely that women with higher values of UDI-6, POPDI-6, and CRADI-8 would have higher values of UIQ-7, POPIQ-7, and CRAIQ-7, respectively; that women who had one or more vaginal childbirths would score higher on the PFDI-20, as this type of childbirth is associated with a higher likelihood of pelvic dysfunctions and also that women who had undergone obstetric surgery would exhibit more symptoms of PFD and a greater impact of these on their quality of life compared to those who were not subjected to such surgery [25].

#### 2.2.4. Statistical Analysis

The statistical treatment of the data obtained was carried out using specialized software: IBM SPSS Statistics version 28. Reliability was assessed through internal consistency. Internal consistency was measured using Cronbach’s Alpha, with values above 0.70 indicating good reliability [23].

For sociodemographic and clinical characterization, as well as questionnaire scores, descriptive statistical analysis. Spearman’s correlation (rs) was used, and for the analysis of differences between groups, the T-student test was utilized.

Statistical tests were interpreted using a significance level of 0.05 (*p* ≤ 0.05) and a 95% confidence interval.

## 3. Results

The cross-cultural adaptation of the Portuguese versions of the PFDI-20 and PFIQ-7 achieved a satisfactory level of semantic, conceptual, idiomatic, and equivalence adequacy.

### 3.1. Cultural and Linguistic Adaptation

The translation was carried out as planned, and no issues were encountered during the process. The content validity of the Portuguese adaptations of PFDI-20 and PFIQ-7 was assured through collaborative revisions involving experts and the women who participated in the pilot study. During the pilot study, all the women demonstrated a clear understanding of the questionnaire items. No words or questions presented difficulties in comprehension, and no adaptations were needed. All participants responded to all items in the Portuguese versions of the PFDI-20 and PFIQ-7 questionnaires, and no missing items were found. The structure of the original versions remains intact in the final Portuguese adaptations of the PFDI-20 and PFIQ-7 questionnaires.

### 3.2. Psychometric Validation of the Portuguese Versions of the PFDI-20 and PFIQ-7

#### 3.2.1. Sample

From the 305 women who participated in the questionnaire, only 287 (94.1%) met the eligibility criteria for inclusion due to inconsistencies in their responses. The mean age of the sample was 33.47 ± 8.2 years (Table 1).

Among the 287 participants, 23% had an educational level equal to or below the 12th grade, while the majority of the sample held at least a university degree. Regarding obstetric history, 161 participants (56.1%) had undergone at least one childbirth, with 13.6% of these being cesarean deliveries and 42.5% being vaginal births. Only 2.4% had undergone a hysterectomy, and 18.8% had undergone other obstetric surgeries. Of the total population, 73 (25.4%) had a history of UI, 10 (3.5%) reported a history of FI, and 30 (10.5%) reported a history of POP.

#### 3.2.2. Reliability

Reliability results assessed through internal consistency are described in Table 2.

The Portuguese versions demonstrated strong internal consistency. For the PFDI-20, Cronbach’s alpha was 0.85, with subscale values of 0.71 for POPDI-6, 0.74 for CRADI-8, and 0.81 for UDI-6. For the PFIQ-7, Cronbach’s alpha was 0.94, with subscale values of 0.92 for UIQ-7, CRAIQ-7, and POPIQ-7. All values were statistically significant (*p* < 0.001).

#### 3.2.3. Content Validity

Content validity was established through a review conducted by a multidisciplinary panel of experts during the adaptation process and qualitative analysis of feedback from the population during the pilot study. The expert panel reached a consensus that the questionnaires contained all the pertinent questions, and no supplementary questions were incorporated beyond those in the original versions. The target population indicated that the questionnaires were well understood and readable. No Ceiling Effect was observed in either of the Portuguese versions: POPDI-6 (0%); CRAD-8 (0%); PFDI-20 (0%); UIQ-7 (0%); CRAIQ-7 (0%); POPIQ-7 (0%); PFIQ-7 (0%), except for the UDI-6 subscale, which showed a ceiling effect of 0.7%. However, a Floor Effect was identified in both Portuguese versions of the PFDI-20 (5.2%), POPDI-6 (36.6%), CRAD-8 (20.2%), UDI-6 (16%) and PFIQ-7 (41.5%), UIQ-7 (59.9%), CRAIQ-7 (69%), POPIQ-7 (72%).

#### 3.2.4. Criterion Validity

The comparison of the questionnaires (PFDI-20 and PFIQ-7) among themselves and with the five questions (Q1–Q5), assessed through Spearman correlation, is presented in Table 3.

In Table 3 the criterion validity was high and confirmed by examining the correlations of the Portuguese versions of the PFDI-20 and PFIQ-7 and their respective subscales with questions Q1–Q5. This indicates a significant impact of POP on the quality of life and the overall perception of health. The highest Spearman coefficients were both related to the correlations of the Portuguese version of PFIQ-7 with Q2 and Q3. Construct validity was further confirmed by the correlation between the PFDI-20 and PFIQ-7, rs = 0.652 (*p* < 0.01).

#### 3.2.5. Construct Validity

All predefined hypotheses were confirmed, as shown in Table 3 and Table 4.

Construct validity was established through the comparison of women who underwent vaginal childbirth with those who did not. A greater prevalence of PFD symptoms was observed among women who had experienced vaginal childbirth, in comparison to those who had not. The POPDI-6 and UDI-6 subscales of the Portuguese version of the PFDI-20 questionnaire are the only ones that show significance (*p* = 0.04). In the history of obstetric surgery, all correlations were significant (*p* < 0.05). Higher values are demonstrated regarding PFD symptoms and their impact on the quality of life in women who underwent this type of surgery when compared to those who were not subjected to it. These data are higher in the UDI-6 (31.48 ± 22.93) and CRAIQ-7 (17.27 ± 25.81) subscales (Table 4).

## 4. Discussion

The PFDI-20 and PFIQ-7 questionnaires are strongly recommended as essential instruments for assessing pelvic floor disorder symptoms and quantifying their impact on the quality of life among the studied population [26]. Both the PFDI-20 and PFIQ-7 have been translated into several languages, including Hispanic Spanish, Amharic, French, Swedish, Arabic, Brazilian Portuguese, Finnish, and Dutch, among others [20,24,27,28,29,30]. However, they had not yet been validated for the Portuguese language. Within this study, the reliability and the validity of the Portuguese adaptations of the PFDI-20 and PFIQ-7 questionnaires were assessed. These instruments were successfully translated and culturally adapted to the European Portuguese context. The results demonstrated excellent internal consistency, strong criterion validity, and good construct validity. These findings are consistent with validations of other versions, such as the Spanish and Amharic versions [20,24,27,28,29,30]. All predefined hypotheses were confirmed.

For an instrument to be used effectively in a specific population, it must undergo a thorough validation process, including linguistic, cultural, and psychometric validation. In terms of linguistic and cross-cultural adaptation, maintaining the original context, meaning, instructions, and presentation of the questionnaire is essential to ensure semantic, conceptual, and operational equivalence. The translation processes for the European Portuguese versions of the PFDI-20 and PFIQ-7 were conducted following the same procedures as previous validations in Amharic, Spanish, Finnish, Brazilian Portuguese, and Swedish [19,20]. Content validity was confirmed through expert review, independent bilingual translation, and pilot testing, revealing no incongruities and aligning with the findings of other validation studies [19,20]. These steps ensure that the instrument is not only linguistically accurate but also culturally appropriate, supporting its reliable use in clinical settings within European Portuguese-speaking populations.

As anticipated, the reliability of the Portuguese versions of the PFDI-20 and PFIQ-7 was confirmed. Similar values are found in the Amharic, Spanish, Finnish, Brazilian Portuguese, and Swedish versions [20,24,27,28,29,30].

While there is no universally acknowledged gold standard for pelvic floor disorder symptoms, we relied on the correlation between PFDI-20, PFIQ-7, and Q1–Q5 to estimate criterion validity. It is noteworthy that the criterion validity falls within an acceptable range. These outcomes are consistent with prior studies, even though they employed distinct criterion standards [23].

No ceiling effects were observed for any of the Portuguese versions, except for the UDI-6 subscale, which presented a negligible ceiling effect of 0.7%. Conversely, a marked floor effect was identified in both Portuguese versions, particularly for the PFIQ-7 (41.5%) and its subscales (UIQ-7: 59.9%; CRAIQ-7: 69%; POPIQ-7: 72%), exceeding the recommended psychometric threshold of 15%. This outcome likely reflects the predominance of asymptomatic participants in the sample (UI: 25.4%; FI: 3.5%; POP: 10.5%), rather than a limitation of the instrument itself, as most respondents reported no impact on quality of life. In contrast, all PFDI-20 subscales showed floor effects below 15%, indicating satisfactory score distribution and supporting their sensitivity and validity for assessing symptom presence and severity in this population.

The results of construct validity indicate a strong association between Q1, Q2, Q3, Q4, Q5 and the Portuguese versions of the PFDI-20 and PFIQ-7, suggesting that questions related to both physical fitness perception and mental well-being significantly impact the lives of the studied population. A significant correlation was also established between the Portuguese versions of the PFDI-20 and PFIQ-7 and the history of obstetric surgery. The lack of a significant association between PFIQ-7 scores and vaginal childbirth history was not unexpected. The PFIQ-7 measures the impact of pelvic floor symptoms on quality of life rather than symptom presence or severity. Although vaginal childbirth is a known risk factor for pelvic floor dysfunction, previous studies suggest that the subjective impact on daily life depends on factors such as coping strategies, health perception, and psychosocial context. Therefore, the PFIQ-7 may not strongly correlate with delivery mode, consistent with the previous literature [31]. Despite the good correlation, it would have been expected to observe a more substantial difference between women who did not undergo childbirth versus those who had a vaginal childbirth and that the difference in PFD symptoms would be more pronounced in the CRADI-8 subscale between the two groups. This might be attributed to the limited sample size (*n* = 10) of individuals with fecal incontinence.

Through our analysis, we can conclude that the incidence of PFD is higher in women with a level of education equal to or lower than the 12th grade, suggesting potential difficulties in accessing literacy and/or healthcare. In future evaluations, it would be interesting to explore geographical differences in academic background to investigate any correlation with PFD, as well as analyze the socioeconomic status of the target population.

As a limitation, we acknowledge that the validation process could have been supported by another gold standard for criterion validity. Additionally, we did not perform a test–retest analysis to assess temporal stability, which is an important component of reliability in PROM validation. This omission means that the stability and reproducibility of the instrument’s scores over time cannot be confirmed, limiting the strength of conclusions regarding measurement consistency [31]. Moreover, the high floor effect observed in the PFIQ-7 represents a limitation of the present study, suggesting that future investigations should include samples with a higher prevalence of UI, FI, and POP to better explore the instrument’s responsiveness and variability across different severity levels. Our sample, although relatively large and exceeding the sample sizes of most previous validations for these instruments, was drawn from clinical settings, which may limit the generalizability to other settings or populations within Portugal. Based on these limitations, we recommend that future studies include a test–retest component to further evaluate score stability over time and utilize an established gold-standard instrument for criterion validity if available. Expanding recruitment to more diverse and representative samples—including women from rural, institutionalized, or different socioeconomic backgrounds—would strengthen the external validity of future research.

Nevertheless, it is important to note that these short forms can be readily incorporated into clinical practice as routine screening tools or during follow-up visits. Their use allows for the quantification of symptom severity and quality of life impact, thereby guiding personalized management and supporting shared decision-making in women with PFD. The adoption of the PFDI-20 and PFIQ-7 enables clinicians to objectively track symptom progression and treatment response, underpinning informed care decisions and optimizing patient outcomes.

## 5. Conclusions

The validated short versions of the PFDI-20 and PFIQ-7 in European Portuguese have achieved semantic, conceptual, idiomatic, and content equivalence with the original versions. Both instruments exhibit strong reliability and validity in the evaluation of symptoms and the quality of life in women with PFD. These instruments provide a robust tool for both clinical assessment and cross-cultural research, supporting standardized evaluation of PFD in Portuguese-speaking populations.

## Figures and Tables

**Table 1:** Sociodemographic and Clinical Characteristics of the Study Population.

Characteristic	
Age (years) Mean ± SD	33.47 ± 8.2
Formal Education n (%)	
Up to 12th Grade	66 (23%)
Higher Education	154 (53.7%)
Master’s Degree	63 (22%)
Doctorate	4 (1.4%)
BMI Mean ± SD	23.67 ± 3.8
Childbirths n (%)	
Yes	161 (56.1%)
No	126 (43.9%)
Number of Childbirths n (%)	
1	92 (32.1%)
2	58 (20.2%)
3	10 (3.5%)
4	1 (0.3%)
How Long Ago? n (%)	
Less than 12 Months	47 (16.4%)
More than 12 Months	114 (39.7%)
Type of Childbirth n (%)	
Caesarean Section	39 (13.6%)
Vaginal	122 (42.5%)
Hysterectomy n (%)	
No	280 (97.6%)
Yes	7 (2.4%)
Other Obstetric Surgery History n (%)	
No	233 (81.2%)
Yes	54 (18.8%)
Urinary Incontinence History n (%)	
No	214 (74.6%)
Yes	73 (25.4%)
Fecal Incontinence History n (%)	
No	277 (96.5%)
Yes	10 (3.5%)
Prolapse History n (%)	
No	257 (89.5%)
Yes	30 (10.5%)
Menopause n (%)	
No	268 (93.4%)
Yes	15 (5.2%)
Don’t know	4 (1.4%)

Abbreviations: BMI: Body Mass Index (kg/m^2^); SD: standard deviation.

**Table 2:** Internal Consistency of Portuguese Versions of the PFDI-20 and PFIQ-7 Questionnaires.

Internal Consistency (*n* = 287)	
Cronbach’s Alpha	Cronbach’s Alpha
PFDI-20 = 0.85	PFIQ-7 = 0.94
POPDI-6 = 0.71	UIQ-7 = 0.92
CRADI-8 = 0.74	CRAIQ-7 = 0.92
UDI-6 = 0.81	POPIQ-7 = 0.92

Abbreviations: PFDI-20: Pelvic Floor Distress Inventory; POPDI-6: Pelvic Organ Prolapse Distress Inventory; CRADI-8: Colo-rectal-anal Distress Inventory; UDI-6: Urinary Distress Inventory; PFIQ-7: Pelvic Floor Impact Questionnaire; UIQ-7: Urinary Impact Questionnaire; CRAIQ-7: Colo-rectal-anal Impact Questionnaire; POPIQ-7: Pelvic Organ Prolapse Impact Questionnaire.

**Table 3:** Construct validity results (Spearman’s coefficient (rs))—correlation between the instruments and Q1, Q2, Q3, Q4, Q5.

	PFDI-20	POPDI-6	CRADI-8	UDI-6	PFIQ-7	UIQ-7	CRAIQ-7	POPIQ-7
PFDI-20	-							
POPDI-6	0.782 **	-						
CRADI-8	0.680 **	0.412 **	-					
UDI-6	0.858 **	0.555 **	0.346 **	-				
PFIQ-7	0.652 **	0.505 **	0.441 **	0.578 **	-			
UIQ-7	0.584 **	0.434 **	0.260 **	0.648 **	0.787 **	-		
CRAIQ-7	0.419 **	0.320 **	0.494 **	0.238 **	0.674 **	0.353 **	-	
POPIQ-7	0.458 **	0.460 **	0.273 **	0.385 **	0.678 **	0.420 **	0.418 **	-
Q1	0.438 **	0.358 **	0.335 **	0.374 **	0.413 **	0.350 **	0.260 **	0.344 **
Q2	−0.420 **	−0.277 **	−0.336 **	−0.391 **	−0.461 **	−0.366 **	−0.301 **	−0.397 **
Q3	−0.423 **	−0.369 **	−0.270 **	−0.363 **	−0.486 **	−0.399 **	−0.328 **	−0.455 **
Q4	−0.248 **	−0.220 **	−0.231 **	−0.179 **	−0.315 **	−0.207 **	−0.276 **	−0.275 **
Q5	−0.297 **	−0.240 **	−0.311 **	−0.214 **	−0.366 **	−0.273 **	−0.308 **	−0.318 **
	Q1	Q2	Q3	Q4	Q5			
Q1	-							
Q2	−0.396 **	-						
Q3	−0.457 **	0.374 **	-					
Q4	−0.347 **	0.390 **	0.436 **	-				
Q5	−0.360 **	0.365 **	0.376 **	0.443 **	-			

Abbreviations: PFDI-20: Pelvic Floor Distress Inventory; POPDI-6: Pelvic Organ Prolapse Distress Inventory; CRADI-8: Colo-rectal-anal Distress Inventory; UDI-6: Urinary Distress Inventory; PFIQ-7: Pelvic Floor Impact Questionnaire; UIQ-7: Urinary Impact Questionnaire; CRAIQ-7: Colo-rectal-anal Impact Questionnaire; POPIQ-7: Pelvic Organ Prolapse Impact Questionnaire; Q1: Question 1; Q2: Question 2; Q3: Question 3; Q4: Question 4; Q5: Question 5; ** *p* < 0.001.

**Table 4:** Construct validity values between history of childbirth and history of obstetric surgery and the Portuguese versions of the PFDI-20 and PFIQ-7.

	Total Sample (*n* = 287) M ± SD	Vaginal Childbirth History: No (*n* = 126); Yes (*n* = 122)	M ± SD	*p*	Obstetric Surgery History: No (*n* = 233); Yes (*n* = 54)	M ± SD	*p*
PFDI-20	49.44 ± 39.85	No	41.96 ± 36.84	0.007	No	44.43 ± 35.42	<0.001
		Yes	55.45 ± 41.36		Yes	71.01 ± 48.90	
POPDI-6	12.18 ± 15.06	No	9.56 ± 13.04	0.04	No	10.59 ± 12.70	0.007
		Yes	14.79 ± 15.39		Yes	19.06 ± 21.41	
CRADI-8	14.91 ± 14.07	No	13.61 ± 11.63	0.648	No	13.60 ± 13.14	0.003
		Yes	14.40 ± 15.01		Yes	20.54 ± 16.52	
UDI-6	22.36 ± 20.57	No	18.78 ± 18.96	0.04	No	20.24 ± 19.43	<0.001
		Yes	26.37 ± 21.96		Yes	31.48 ± 22.93	
PFIQ-7	23.21 ± 39.98	No	20.54 ± 37.49	0.238	No	17.31 ± 31.18	0.012
		Yes	26.55 ± 42.46		Yes	48.63 ± 59.66	
UIQ-7	9.61 ± 18.12	No	7.48 ± 15.91	0.30	No	8.00 ± 16.37	0.006
		Yes	12.56 ± 20.47		Yes	16.56 ± 23.17	
CRAIQ-7	6.96 ± 15.67	No	5.81 ± 11.45	0.385	No	4.57 ± 10.95	<0.001
		Yes	7.49 ± 18.17		Yes	17.27 ± 25.81	
POPIQ-7	6.63 ± 15.49	No	7.25 ± 15.51	0.356	No	4.74 ± 13.05	<0.001
		Yes	6.51 ± 15.94		Yes	14.80 ± 21.57	

Abbreviations: PFDI-20: Pelvic Floor Distress Inventory; POPDI-6: Pelvic Organ Prolapse Distress Inventory; CRADI-8: Colo-rectal-anal Distress Inventory; UDI-6: Urinary Distress Inventory; PFIQ-7: Pelvic Floor Impact Questionnaire; UIQ-7: Urinary Impact Questionnaire; CRAIQ-7: Colo-rectal-anal Impact Questionnaire; POPIQ-7: Pelvic Organ Prolapse Impact Questionnaire; M: Mean; SD: standard deviation.

## Data Availability

The original contributions presented in this study are included in the article. Further inquiries can be directed to the corresponding author.

## Abbreviations

The following abbreviations are used in this manuscript:

PFDI-20	Pelvic Floor Distress Inventory
POPDI-6	Pelvic Organ Prolapse Distress Inventory
CRADI-8	Colo-rectal-anal Distress Inventory
UDI-6	Urinary Distress Inventory
PFIQ-7	Pelvic Floor Impact Questionnaire
UIQ-7	Urinary Impact Questionnaire
CRAIQ-7	Colo-rectal-anal Impact Questionnaire
POPIQ-7	Pelvic Organ Prolapse Impact Questionnaire
PFMD	Pelvic Floor Muscle Dysfunctions
PFD	Pelvic Floor Dysfunctions
POP	Pelvic Organ Prolapse
FI	Fecal Incontinence
UI	Urinary Incontinence
COSMIN	Consensus-based Standards for the Selection of Health Measurement Instruments
CEIPC	Ethics Committee of the Polytechnic Institute of Coimbra
rs	Spearman’s correlation
SD	Standard Deviation
